# An integrated hospital-district evaluation for communicable diseases in low/middle-income countries

**DOI:** 10.1093/eurpub/ckac131.244

**Published:** 2022-10-25

**Authors:** L Tavoschi, P Belardi, S Mazzilli, F Manenti, G Pellizer, D Abebe, G Azzimonti, JB Nsubuga, G Dall'Oglio, M Vainieri

**Affiliations:** 1 Department of Translational Research in Medicine, University of Pisa, Pisa, Italy; 2 Institute of Management and Department EMbeDS, Sant'Anna School of Advances Studies, Pisa, Italy; 3 Scuola Normale Superiore, Pisa, Italy; 4 Doctors with Africa CUAMM, Padua, Italy; 5 Doctors with Africa CUAMM, Wolisso, Ethiopia; 6 Doctors with Africa CUAMM, Iringa, Tanzania; 7 Doctors with Africa CUAMM, Matany, Uganda; 8 Doctors with Africa CUAMM, Gulu, Uganda

## Abstract

**Background:**

The last two decades saw an extensive effort to design and implement integrated and multidimensional healthcare evaluation systems in high-income countries. However, in low/middle-income countries, few experiences of such systems implementation have been reported in the scientific literature. We developed and piloted an innovative tool to assess the performance of health services provision for communicable diseases in three African countries.

**Methods:**

A total of 42 indicators, 14 per each communicable disease care pathway (Tuberculosis, Gastroenteritis, and HIV/AIDS), were developed. A sub-set of 23 indicators was included in the evaluation process. The indicators assessed four care phases: prevention, diagnosis, treatment, and outcome. All indicators were calculated for the period 2017-2019, while performance evaluation was performed for 2019. The analysis involved four health districts and their relative hospitals in Ethiopia, Tanzania, and Uganda.

**Results:**

Substantial variability was observed over time and across the four different districts. In the TB pathway, the majority of indicators scored below the standards and below-average performance was mainly reported for prevention and diagnosis phases. Along the Gastroenteritis pathway, excellent performance was instead evaluated for most indicators and the highest scores were reported in prevention and treatment phases. The HIV/AIDS pathway indicators related to screening and outcome phases were below the average score, while good or excellent performance was registered within the treatment phase.

**Conclusions:**

The bottom-up approach and stakeholders’ engagement increased local ownership of the process and the likelihood that findings will inform health services performance and quality of care. Despite the intrinsic limitations of data sources, this framework may contribute to promoting good governance, performance evaluation and accountability in settings characterised by multiple healthcare service providers.

**Key messages:**

• A successful experience in developing and implementing a communicable diseases performance evaluation systems in three sub-Saharan African countries using a bottom-up approach.

• The communicable diseases performance evaluation tool helped the data sharing between local healthcare providers and the development of competencies in data collection, analysis and interpretation.

